# MPrESS: An R-Package for Accurately Predicting Power for Comparisons of 16S rRNA Microbiome Taxa Distributions including Simulation by Dirichlet Mixture Modeling

**DOI:** 10.3390/microorganisms11051166

**Published:** 2023-04-29

**Authors:** Thomas H. Clarke, Chris Greco, Lauren Brinkac, Karen E. Nelson, Harinder Singh

**Affiliations:** 1 J. Craig Venter Institute, 9605 Medical Center Drive, Suite #150, Rockville, MD 20850, USA; 2 Noblis, Reston, VA 20191, USA

**Keywords:** dirichlet mixture modeling, 16S rRNA gene sequencing, power calculations, sample size estimates, human microbiome, forensics

## Abstract

Deep sequencing has revealed that the 16S rRNA gene composition of the human microbiome can vary between populations. However, when existing data are insufficient to address the desired study questions due to limited sample sizes, Dirichlet mixture modeling (DMM) can simulate 16S rRNA gene predictions from experimental microbiome data. We examined the extent to which simulated 16S rRNA gene microbiome data can accurately reflect the diversity within that identified from experimental data and calculate the power. Even when experimental and simulated datasets differed by less than 10%, simulation by DMM consistently overestimates power, except when using only highly discriminating taxa. Admixtures of DMM with experimental data performed poorly compared to pure simulation and did not show the same correlation with experimental data *p*-value and power values. While multiple replications of random sampling remain the favored method of determining the power, when the estimated sample size required to achieve a certain power exceeds the sample number, then simulated samples based on DMM can be used. We introduce an R-Package, MPrESS, to assist in power calculation and sample size estimation for a 16S rRNA gene microbiome dataset to detect a difference between populations. MPrESS can be downloaded from GitHub.

## 1. Introduction

The human microbiome is composed of communities of microorganisms, including bacteria, that live on and in the human body. These bacteria form distinct ecologies that can be explored through targeted deep sequencing and subsequent grouping of its community members through operational taxonomic units (OTUs). By cataloging the OTUs, through counts of mapped sequencing reads, microbial communities can be correlated with personal identity [1,2], geographic location [3], human body site [4], and health status of an individual [5], enabling the microbiome to be used to investigate forensic questions [1,2,6,7,8]. Furthermore, it may eventually be possible to address forensic questions about a subject based solely on their microbiome [8], as microbiomes from distinct populations can be differentiated, such as those that maintain a particular lifestyle such as smoking [9] or diet [10]; or populations with or without a disease [11,12,13]; or populations from different geographic locations [3,14].

There are several analysis techniques that can be used to differentiate microbiomes. One involves an examination of the total OTU table, such as with permutational multivariate analysis of variance (PERMANOVA). Alternatively, the distance between sets of taxa, either with a phylogenetically-dependent metric such as UniFrac [15] or a phylogenetically-independent metric such as Bray-Curtis [10], can be used. Microbiomes can also be discriminated by identifying taxa that are significantly different between the populations using algorithms in various packages such as DESeq2 [9]. As these methods use differences between microbiome data (OTU tables), they require a sufficient number of samples to permit the observation of statistically significant differences between different groups [15]. To test a hypothesis, it is often first necessary to calculate the statistical power of the studied microbiomes by identifying the capacity of microbiome OTUs to discriminate different metadata variables across different sample sizes [16,17]. The statisical power calculation (power) gives the number of samples needed to detect the differences at a user supplied cutoff, and can be used by researchers to design the microbiome sample collection to fully address the questions the study wishes to addess. To calculate power from existing OTU tables, multiple programs employing a variety of techniques are currently available, some of which can also expand the original sample number by supplementation with simulated data [18,19,20]. One example, Micropower, randomly subsamples with replacement from the different populations before determining the differences with PERMANOVA analysis [18]. Another existing R module, MicrobiomeDASim, uses only simulated data from user-given parameters to calculate the power necessary to successfully discriminate taxa [21]. MicrobiomeDASim permits researchers to examine multiple previously unloaded data, such as looking at statistical tests not previously used and estimating different time points for longitudinal studies, but it does require the user to estimate the parameter space expected for the microbiomes. Alternatively, the R module powmic calculates the power similarly using a synthetic microbiome OTU table calculated based on estimates by the user [22].

Beyond either sampling or using simulated data from user-supplied estimations, OTU tables can be approximated by simulation from the initial dataset. Dirichlet mixture modeling (DMM) is one of the most widely utilized methods [23,24,25,26]. DMMs allow for multivariate, oversampled data where the majority of taxa are represented by only a few or no reads, and a minority of taxa contain the majority of the reads, which is the distribution pattern of most OTU tables. DMM models therefore can use existing OTU tables to estimate parameters, and, from the parameters, stochastically generate simulated OTU tables based on the models, as is achieved in the HMP R package [20]. One available program that uses the HMP R package-based DMM to perform OTU table power calculations is the web application described in Mattiello et al. This uses Wald-test-based power calculations to determine the differences between the populations and a priori knowledge of the shape and other parameters of the desired OTU tables as the default [19]. It is also possible to calculate power to identify discriminating taxa, as can be performed using the web-portal program discussed prior to using the Mann–Whitney non-parametric test.

With the availability of these models and programs, we examined the capacity of simulated OTU tables based on models to accurately reflect the diversity within the OTU tables and thus the accuracy of the resulting power calculations. The results of our investigations in combination with R-scripts written to generate and analyze the simulation and sampling datasets led us to create an R-package named MPrESS that calculates the power for pre-existing microbiome sets with distinct metadata values using the best practices discussed below, while also allowing for some user-based specification of the parameters as necessary. Creating an R-package both enables the use of pre-existing microbiome analysis tools, such PERMANOVA and DESeq2, and enables users to use a program that is platform agnostic. Additionally, the use of an R-package allows for a diversity in user inputs which can be used to best model the power. Potential user-specified changes include using only the most discriminating taxa in the provided OTUs; the techniques used to expand the provided dataset; and the cut-offs used to determine the minimal power. The MPrESS package also provides the summary and plotting of the results to enable additional researcher engagement with the data.

## 2. Implementation

### 2.1. MPrESS: Microbiome Power Estimates Using Sampling and Simulation

We created MPrESS, an R-package that predicts the minimum number of samples required to discriminate individuals or groups having different metadata variables with sufficient power and is based on our analyses for the optimal calculations for the datasets, as discussed in the results section. A flowchart showing the MPrESS usage is shown in Figure S1. MPrESS uses 16S rRNA gene microbiome data with the associated metadata as a Phyloseq-class object [27]. It allows for user input for multiple variables that are used in the power calculation, such as the following: alpha and beta-errors (default values which are 0.05 and 95%, respectively); the metadata variable and values to compare; the distance metric and statistical test to use; the number of replicates to run including the minimal number to start off with; and whether to only use differentially abundant taxa or all the available taxa. The MPrESS package prioritizes power calculations using only subsampled data, but once the sample number necessary to reach the desired power exceeds the number of samples available for a given metadata variable, the package switches to only using simulated data. We found that combining sampled data with simulated datasets performs worse than pure simulated datasets in mirroring the sampled data as compared with PERMANOVA. Thus, only fully simulated datasets are used by MPrESS when extending the sample numbers, which is required to calculate the power.

To calculate the necessary power, MPrESS either samples or simulates tables from the initial OTU data (Additional File S1: Figure S1). The sampled data are obtained by using the R base sampling function to randomly select the OTU samples without replacement from the initial data. The simulated OTU tables are made using the Dirichlet multinomial function in the HMP package [20]. For the DMM, the gamma shapes for each metadata value are estimated from the original OTUs data for a particular metadata variable. The number of reads per sample is calculated from the original data by sampling with replacement. The same shape distribution is used across the multiple replicates for the simulation calculations. The difference between the OTUs data is calculated using PERMANOVA, with either phylogenetically aware (UniFrac) or raw (Bray–Curtis) distances as specified by the user, using the vegan and phytools R packages, respectively [28,29].

Additionally, power calculations can also be directed to be performed only using the OTUs that are the most discriminating between a given metadata variable. In these instances, DESeq2 is used by MPRESS with the either the FDR-corrected *p*-value cutoff specified or the number of taxa to be used as given by the user to identify the discriminating taxa. The OTUs raw counts are normalized and compared using the negative binomial distributions according to the DESeq2 default parameters [30]. The OTUs table is then trimmed to only include significant discriminating taxa determined by DESeq2 and the rest of the calculation pipeline is the same as when analyzing full OTU tables.

**Figure 1:**
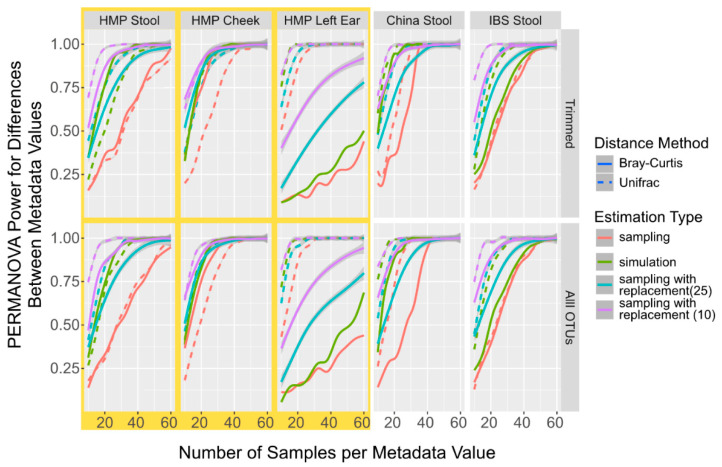
Power Calculation to Detect Population Differences with PERMANOVA in Simulated and Sampled Data. Smoothed lines showing the mean power to detect differences between different populations with PERMANOVA sampled without replacement (red), sampled with replacement using 25 initial samples (light blue), sampled with replacement using 10 initial samples (purple), and simulated (green) microbiome datasets at different sample numbers and with different distances metrics, Bray–Curtis (solid line) and UniFrac (dotted line).

After the power values are calculated, the package returns a MPrESS class object that contains all the power calculation information including the sample number required to perform the power calculation and the final estimation type. Additional information about the estimations and the tests are shown in a table with OTUs profile information at each sample number, which can be accessed by printing or plotting the MPrESS object. The default MPrESS object plot is created with ggplot and shows the mean *p*-value and the estimated discriminatory power at each run as a smoothed line with the individual means shown as distinct points [31]. An example of the plot is shown in Figure 1.

### 2.2. Microbiome Datasets Analyzed

Publicly available 16S rRNA gene microbiome datasets which incorporate samples from various locations, populations, and health status were used to guide and analyze MPrESS power calculations (Additional File S1: Table S1). The 16S rRNA read sequences from V3-V5 variable regions and three of the available body sites were downloaded from the HMP Data Analysis and Coordination Center, http://hmpdacc.org/HM16STR/ (accessed on 6 March 2019) [32]. Metadata for the samples was obtained with permission from dbGAP using the id #phs000228 [33]. These samples were collected from healthy participants mostly residing in two different geographical locations in the United States: Missouri and Texas. The three body sites (stool, cheek, and left ear) were selected for prevalence in the published data, with unpublished analysis showing high geographic specificity, and similarity to our previous research [4]. As the HMP data is from a Western, homogeneous population, we also tested stool samples from a non-Western, heterogeneous, healthy population residing in two regions in China: Yunnan and Guangxi Zhuang [34]. Additionally, we analyzed stool samples from a Spanish population consisting of individuals diagnosed with and without irritable bowel syndrome (IBS) [35], a syndrome strongly correlated with microbiome effects [36,37]. Sequences and metadata for the China and Spanish IBS projects were obtained from MG-RAST [38] using Project ID 1538 and from NCBI BioProject (PRJNA268708), respectively.

### 2.3. 16S rRNA Gene Analysis

The samples downloaded from each study were processed using an in-house 16S rRNA gene data analysis pipeline. OTUs were generated using the default parameters in UPARSE [39] and taxonomies were assigned to these OTUs with mothur [40] using the 123 version of the SILVA 16S rRNA gene database [41] as the reference database. Samples with less than 2000 reads, or OTUs with less than 10 reads, were discarded from the analysis. Datasets containing either all of the high quality OTUs (labeled “All OTUs”) or contained only OTUs with matches to known genera (labeled “Trimmed”) were both examined. All microbiome datasets were loaded into R using Phyloseq [27] with the OTU phylogenies calculated using the UPARSE-derived multiple alignments of the OTUs and the neighbor-joining function from the APE package [42]. We have included both the Spanish IBS and China OTUs table and metadata in the Phyloseq format as the example data for MPrESS. The HMP OTUs and metadata are not included since the HMP dataset includes dbGAP-derived metadata, but documentation on how to obtain and process the data is available on the GitHub site.

## 3. Results and Discussion

### 3.1. Comparison of Body Sites and OTU Tables in the Simulation versus Sampling

We first examined the differences between different microbiome datasets across multiple body sites and metadata values, including geographic location and health status. OTU tables were generated by random sampling subjects without replacement (“sampled”) and by simulating based on the DMM (“simulated”). For the HMP dataset, samples from two states in the United States, Missouri and Texas, were used; for the China data, samples from two regions in China, Yunnan and Guangxi, were compared; and for the IBS dataset, samples marked as healthy and diagnosed with IBS were analyzed. We used the default alpha and beta values of 0.05 and 0.95 which are traditional values used to both detect differences and to be confident that these differences are repeatable. These microbiome datasets had significantly different OTUs from each other when they were compared by PERMANOVA analysis using both UniFrac and Bray–Curtis distances. The distances between sampled and simulated datasets (“extra-”) were significantly increased when compared to the distances between OTU tables generated only from sampling and between OTUs only generated from simulations (“intra-”), as calculated with two-sample *t*-tests (Additional File S1: Figure S2). This was observed across different distance methods (Bray–Curtis and UniFrac) and body sites (stool, oral cavity inner cheek, and left ear skin). For all datasets, the Bray–Curtis distance between extra and intra-dataset samples was more significant than UniFrac (Additional File S1: Figure S1). Despite this significance, the “intra-” and “extra-” dataset calculation differences are still around 10%. Additionally, the different studies showed distinct characteristics, with the oral cavity inner cheek sampled OTU tables having the most similarity compared to the simulated OTU tables and the left ear skin OTU tables showing the greatest divergence.

### 3.2. Simulation of OTU Tables Underestimates the Number of Samples to Reach the Power Calculation

Given that the sampled and simulated derived OTU tables differed by less than 10%, we investigated how the power calculations using PERMANOVA compared between the two approaches, and, if they were different, b = both how consistent the differences were and what some potential influential factors might have been. Since sampling with replacement is used in other available power calculation packages [18], we also calculated power using PERMANOVA from OTU tables generated from sampling with replacement. Since sampling with replacement from a large initial dataset is very similar to sampling without replacement, we used only 10 randomly selected samples per metadata variable per run and only 25 initial samples. Across all datasets, including both the complete and trimmed taxa, the simulation consistently underestimates the number of samples required for 95% of the replications to be significantly different by PERMANOVA when compared to sampling (Figure 1). It was also observed with the data generated using the sampling with the replacement procedure in the initial starting levels, though the tests with the smaller initial set (purple lines in Figure 1) constantly had a higher over-estimation of the power values. The differences are between 7 and 24 samples for simulated data and 1 and 40 samples for data sampled with replacement when starting from 10 samples, and when observable using UniFrac distances. When measuring with the Bray–Curtis distance, the data differences are between 1 and 31 for data generated by sampling with replacement when compared to the data generated by simulating data, and between 2 and 31 samples when compared to the data generated by sampling with replacements (Additional File S1: Table S2). However, the distance method used and body site from where the sampled data were derived are more influential in the final power calculation than sampling versus simulation. While we are not certain about what might be driving this constant underestimation of the numbers of samples required to reach the power, we did observe that the simulated datasets have almost consistently higher alpha diversity than the sampled datasets across all body sites and numbers of taxa (Additional File S1: Figure S3), even as the alpha-diversity differed significantly between the different datasets. We also observed that increasing the sample number had no significant effect on the alpha diversity differences. We hypothesize that the increase in alpha diversity is caused by the increased likelihood of having a non-zero taxa in the simulated samples compared to the experimental samples. Thus, while both simulated sampling and sampling with the replacement procedure can have similar overestimations for the power calculations, sampling with replacements is more divergent once the number of samples necessary to determine the power calculation greatly exceeds the number of available samples. Therefore, for MPrESS, we chose the simulated over sampling with replacement.

### 3.3. Sampling versus Simulating after Identifying Discriminating Taxa with DESeq2

**Figure 2:**
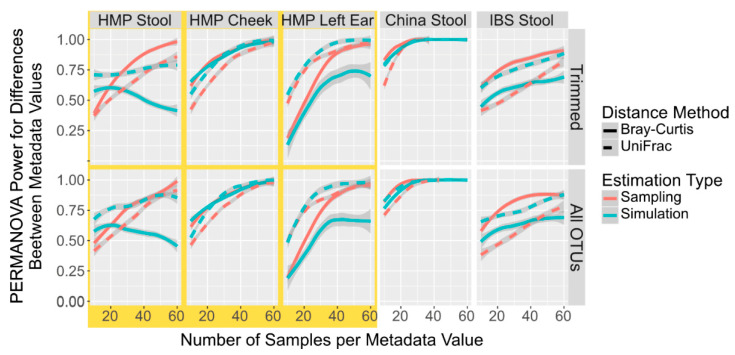
Power Calculation to Detect Population Differences with PERMANOVA in Simulated and Sampled Data With Only Differentially Abundant Taxa. Smoothed lines showing the mean power to detect differences between population locations with PERMANOVA in sampled (red) and simulated (blue) microbiome datasets with only the top 10 most discriminating taxa selected by DESeq2 at different sample numbers and with different distances metrics, Bray–Curtis (solid line) and UniFrac (dotted line).

As microbiome variation attributed to an individual’s traits, lifestyle, health status, and environment can often be concentrated in only a few taxa [43], it is possible that simulations of only these taxa can give a more accurate power calculation than a simulation of the all the taxa. We thus compared the power calculations from sampled and simulated datasets using only these discriminatory taxa as determined by DESeq2. When using an FDR cut-off of 0.05, less than two taxa were returned between 5% and 35% of the time when using 25 samples across the 5 datasets, and thus we were unable to generate PERMANOVA comparisons at those times. Therefore, we used the top 10 most discriminating taxa as determined by the DESeq2 FDR. When comparing simulated and sampled I profiles containing only the top 10 differentially abundant taxa, the HMP Cheek and the China simulated and sampled datasets mirrored each other closely (Figure 2). However, tIOTU profiles simulated from the Spanish IBS Stool, HMP Stool, and Left Ear using only the differentially abundant taxa underrepresent the power compared to the saIed OTU profiles from the same taxa. All of these results differ from the non-DESeq trimmed data (Figure 1), often by giving power values greater than by sampling all OTUs. This suggests that simulated power calculations from differentially abundant taxa can be used without any additional correction since they will return a value very close (though slightly higher). ith the HMP Cheek and the China sample. However, in instances such as the Spanish IBS Stool samples, the simulation gives much higher sample numbers required to reach the power required.

### 3.4. Using Simulation to Extend Small Sample Data

**Figure 3:**
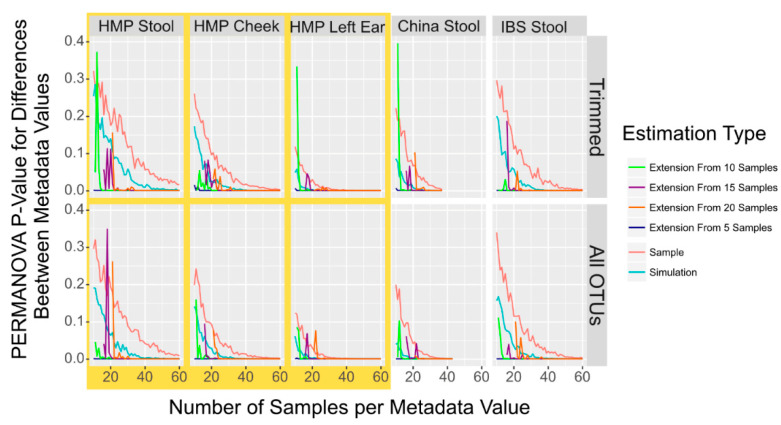
PERMANOVA *p*-Value to Detect Population Differences in Simulated, Sampled, and Combined Sampled and Simulation Data. Smoothed lines showing the mean *p*-value to detect differences between population locations with PERMANOVA in sampled (red) and simulated (blue) microbiome datasets in addition to four sets of combined datasets where sampled microbiome datasets from 5 (dark blue), 10 (green), 15 (purple) or 20 (orange) samples have additional simulated microbiome samples appended. While small variations in the mean *p*-value are indicated by the zagged lines, the trend is cInstant.

Last, we investigated whether extenIg the OTU data with a dataset containing only a small sample size with simulated data would give a more accurate result as compared to a dataset that was constructed only from simulated data. We compared tests of datasets with samples of different starting sizes and then appending data either simulated from the starting samples or using completely sampled and simulated data (Figure 3). While appending only a single simulated sample does fairly accurately reflect the sampled data, we found that appending multiple simulated samples almost always gave a PERMANOVA *p*-value for differences between the samples from the two metadata of <0.001. We wanted to quantify the extent to which simulated data gave a close approximation of the sampled data to verify 7 imulation simultion can accurately be used for power prediction, as seen in Figure 3. We used the Pearson correlation which calculates the extent to which the simulated data match the direction and level of sampled data versus the extended data. We found that simulated data, however, give a lower *p*-value for the power but do mirror the sampled dataset more closely than the extended sampling (simulation vs. sampling: r = 0.92, extension vs. sampling: r = 0.37, Pearson correlation).

It is possible that extending the data by sampling performed poorly solely due to the smaller number of samples used to calculate the DMM model, and that it was not an inherent problem with mixing sampled and simulated data within a dataset. To verify that the decreased PERMANOVA similarity was not driven by sample size, we compared sampled and simulated datasets only derived from a small sample sized dataset. We analyzed 60 randomly sampled HMP stool samples, 30 from Missouri and 30 from Texas. We compared the initial 60 samples, 60 simulated samples, and 60 samples extended by appending 20 simulated OTU profiles to 40 sampled using 100 replicates. For each replicate, a new set of 60 randomized samples was selected. We again observed that the simulated dataset constantly represented the sampled dataset more closely in PERMANOVA significance, as measured by the percent difference in the PERMANOVA *p*-value (extension: 15% vs. simulation: 85%) and in power (extension: 53% correct call vs. simulation: 56%), though the difference was not as pronounced.

## 4. Conclusions

MPrESS is a novel R software package that enables researchers to determine the minimum number of samples required to address a given study hypothesis using 16S rRNA gene microbiome data with sufficient power. MPrESS expands upon existing power calculation programs by integrating both sampling and simulations into the power estimation. Additionally, the user can compute power calculations based only on a subset of DESeq2 identified taxa. We observed that even when sampled and simulated datasets are different by less than 10%, simulation by DMM consistently overestimates the power necessary, though this is to a lesser extent than either admixed or sampling with replacement. This is especially observable when it is necessary to greatly exceed the initial sample number to calculate the power. Therefore, MPrESS prioritizes the useI existing OTU tables, and only when insufficient numbers of samples are available does it stop using the sampling method and instead performs simulations Iestimate the OTU tables. MPrESS does provide statistics for the power calculation for to the simulation estimates as well as the power calculations after the change in estimation method. This can allow for a visual estimate of the degree of power overestimation.

The MPrESS package is designed to be used Ih 16S rRNA gene OTU data as they are the most often studied in microbiome research. However, the modular format of MPrESS allows for the easy addition of metagenomic data for the power calculation. The user can specify any taxonomy rank, i.e., species, genus, family, etc., to perform the power calculations. Likewise, the package requires seed data for the simulation; therefore, some preliminary data are required at this time for all power calculations derived from MPrESS. Even those power estimation programs which are fully simulated, such as *MicrobiomDeSim,* require parameter estimation, preferably calculated from existing datasets [21]. While MPrESS was expressly designed for use in study development to estimate the number of samples required to address a specific hypothesis, it could be used to assist in the quantification of the effect of using different distance metrics, tests, or the number of taxa in determining the differences between populations in existing data.

## Data Availability

All data supporting the results reported in the article are available from: the HMP Data Analysis and Coordination Center, http://hmpdacc.org/HM16STR/ (accessed on 6 March 2019) for the HMP dataset [32]; MG-RAST [38] using Project ID 1538 for the China study [34]; and NCBI BioProject (PRJNA268708) for the Spanish IBS study [35].

## Supplementary Materials

The following supporting information can be downloaded at: https://www.mdpi.com/article/10.3390/microorganisms11051166/s1, Figure S1: Flowchart showing sampling (B,C) and simulation (E,F) schemas from the initial OTU table (A) using all the taxa (B,E) or from only the most differentially abundant taxa as selected by DESeq2 from the initial dataset (C,F). Simulation data derives from the estimation of the underlying distribution of the OTU table as a Gamma Distribution (D); Figure S2: Distance Within and Between Simulated and Sampled OTU Tables at Different Sample Sizes. The China samples are incomplete due to the limited number of samples (Additional file 1: Table S1); Figure S3: Alpha Diversity in Simulated OTU Tables Significantly Higher in All Samples Compared to Sampled OTU Tables; Table S1: Statistical Overview of Sample Sets; Table S2: Alpha Diversity differences between simulated and sampled datasets at the sample number where Power >0.95.

## Availability and Requirements

Project name: MPrESS: Microbiome Power Estimates using Sampling and Simulation; Project homepage: https://github.com/thclarke/MPress (accessed on 24 April 2023); Source-code available at: https://github.com/thclarke/MPress (accessed on 24 April 2023); Operating system(s): Platform independent; Programming language: R (≥3.4.0); Other requirements: None; License: GNU (GPLv2); Any restrictions to use by non-academics: none.

## Abbreviations

OTUs: operational taxonomic units, PERMANOVA: permutational multivariate analysis of variance, DMM: Dirichlet mixture modeling, HMP: Human Microbiome Project, IBS: irritable bowel syndrome, FDR: false discovery rate, MPrESS: microbiome power estimates using sampling and simulation.
