# Supplementary material for: MPrESS: An R-Package for Accurately Predicting Power for Comparisons of 16S rRNA Microbiome Taxa Distributions including Simulation by Dirichlet Mixture Modeling
Source: Microorganisms. 2023 Apr 29;11(5):1166. doi: 10.3390/microorganisms11051166 (PMC10223547; doi:10.3390/microorganisms11051166)
Supplement: Supplementary file 1 [file microorganisms-11-01166-s001.zip › microorganisms-2272368-supplementary.pdf]

Figure S1 Flowchart showing sampling (B,C) and simulation (E,F) schemas from the initial OTU table (A) using all the taxa (B and E) or from only the most differentially abundant taxa as selected by DESeq2 from the initial dataset (C and F). Simulation data derives from the estimation of the underlying distribution of the OTU table as a Gamma Distribution (D).

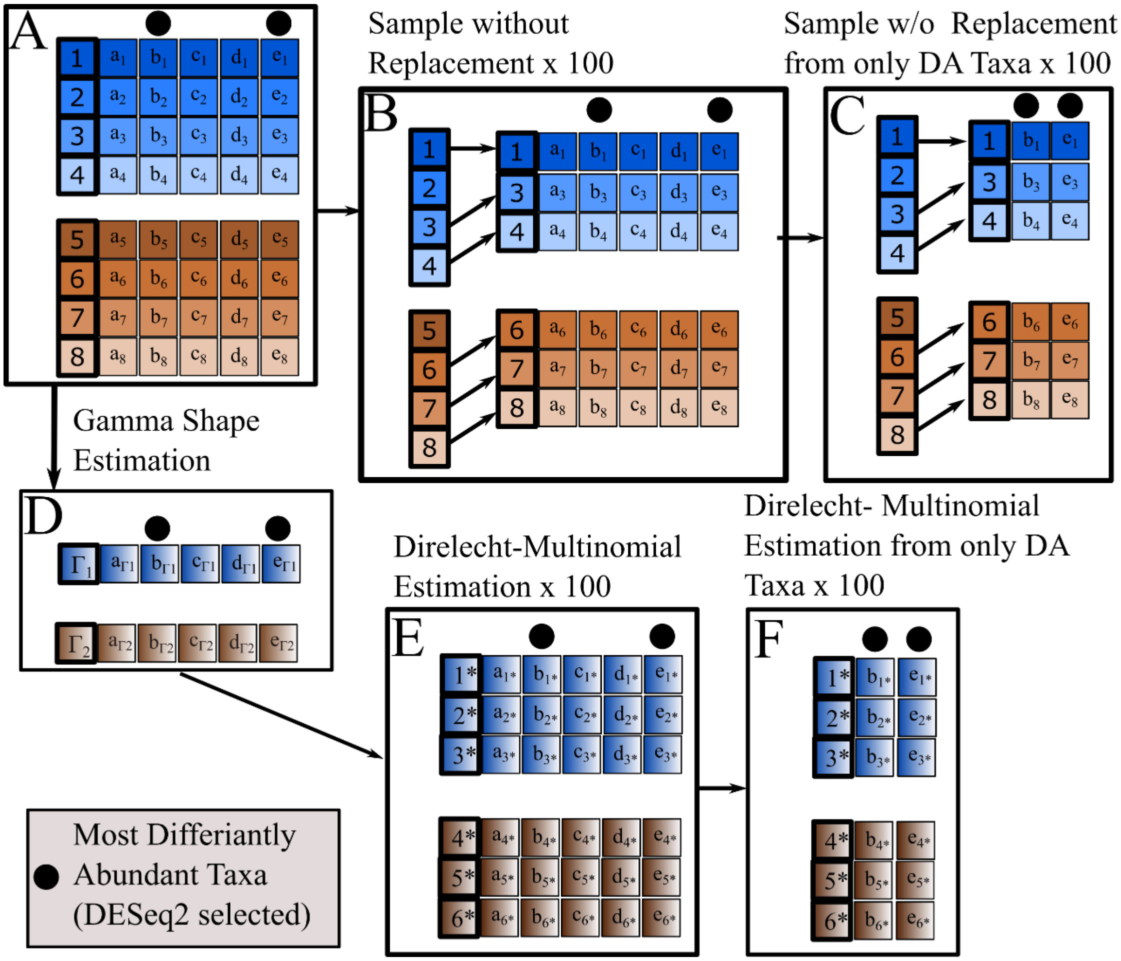

Figure S2. Distance Within and Between Simulated and Sampled OTU Tables at Different Sample Sizes. The China samples are incomplete due to the limited number of samples (Additional file 1: Table S1).

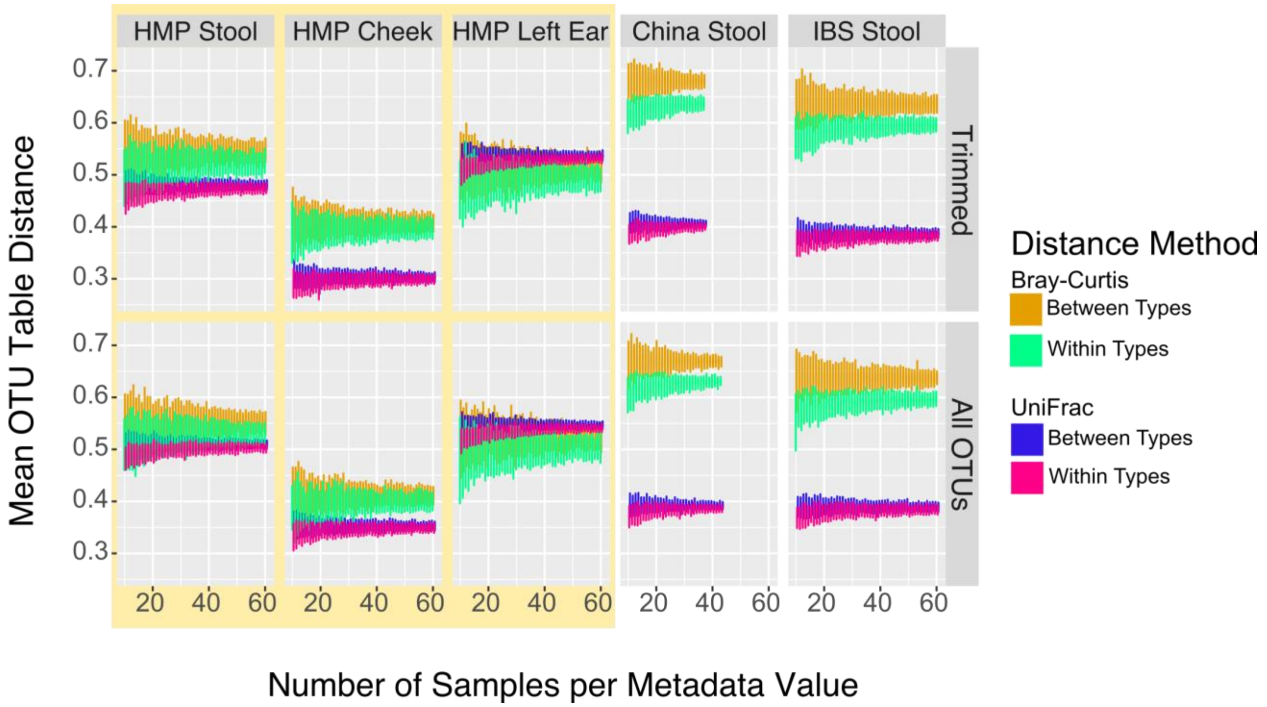

Figure S3. Alpha Diversity in Simulated OTU Tables Significantly Higher in All Samples Compared to Sampled OTU Tables

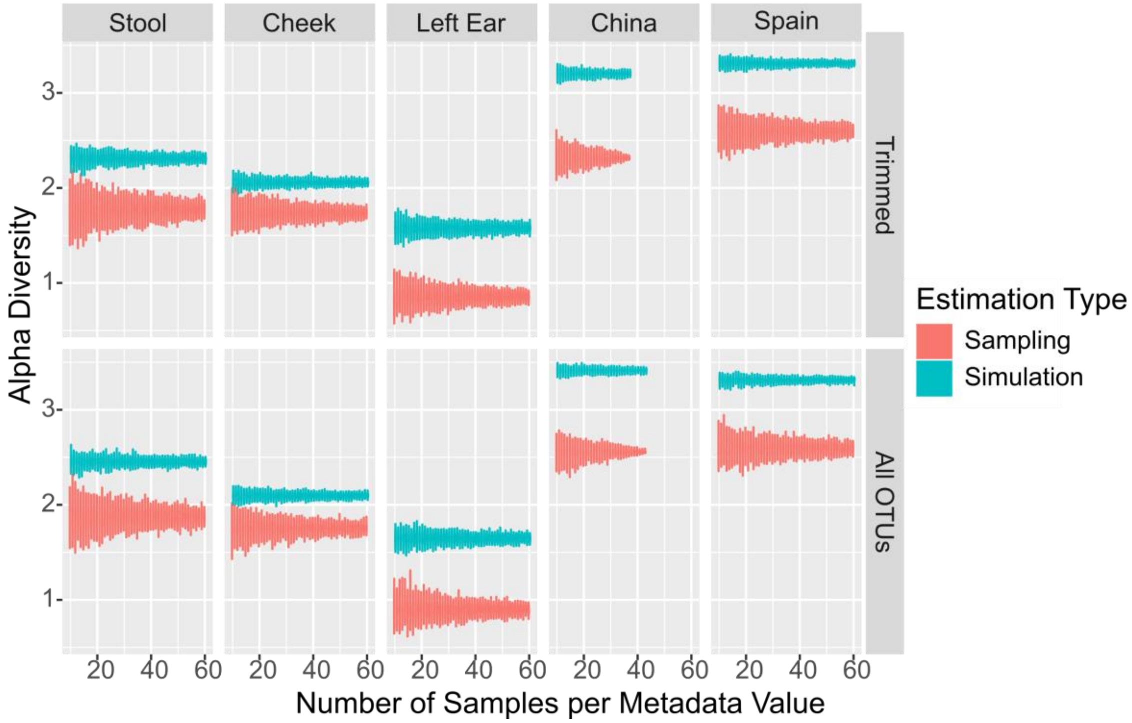

Table S1. Statistical Overview of Sample Sets.

Sample Name	Pubmed ID	Metadata Value 1	Metadata Value 2	Sample Size with Metadata value 1	Sample Size with Metadata value 2	Number of OTUs Full	Number of OTUs Trimmed
HMP Stool	22699609	Texas	Missouri	185	103	203	159
HMP Cheek	22699609	Texas	Missouri	187	88	141	113
HMP Left Ear	22699609	Texas	Missouri	152	86	221	196
China	25647347	Yunnan	Guangxi Zhuang	37	40	255	197
Spain IBS	26239401	Healthy	IBS	199	87	245	211

Table S2. Alpha Diversity differences between simulated and sampled datasets at the sample number where Power > .95:

Dataset	OTUs Used	Distance Metric	Sampling Sample Number	Sampling Alpha Diversity	Simulation Sample Number	Simulation Alpha Diversity	Alpha Diversity Sampling / Simulation
HMP Cheek	Trimmed	Unifrac	27	1.731791	27	2.056525	0.84209
HMP Cheek	Trimmed	Bray-Curtis	27	1.736716	26	2.055036	0.84510
HMP Cheek	Full OTUs	Unifrac	33	1.752964	25	1.754649	0.99904
HMP Cheek	Full OTUs	Bray-Curtis	30	1.76077	25	1.754649	1.00349
HMP Left Ear	Trimmed	Unifrac	24	0.85494	14	1.578455	0.54163
HMP Left Ear	Trimmed	Bray-Curtis	None	None	None	None	N/A
HMP Left Ear	Full OTUs	Unifrac	22	0.90148	15	1.641228	0.54927
HMP Left ear	Full OTUs	Bray-Curtis	None	None	None	None	N/A
HMP Stool	Trimmed	Unifrac	None	None	37	2.307087	N/A
HMP Stool	Trimmed	Bray-Curtis	46	1.77312	27	2.310008	0.76758
HMP Stool	Full OTUs	Unifrac	55	1.872128	31	2.455048	0.76256
HMP Stool	Full OTUs	Bray-Curtis	59	1.879201	28	2.456793	0.76490
China	Trimmed	Unifrac	29	2.30917	17	3.202246	0.721109
China	Trimmed	Bray-Curtis	35	2.313465	19	3.207383	0.721294
China	Full OTUs	Unifrac	27	2.549194	15	3.410471	0.747461
China	Full OTUs	Bray-Curtis	41	2.556264	26	3.416042	0.748312
Spain IBS	Trimmed	Unifrac	44	2.59242	27	3.30835	0.783599
Spain IBS	Trimmed	Bray-Curtis	46	2.595819	44	3.310405	0.784139
Spain IBS	Full OTUs	Unifrac	45	2.594876	29	3.312029	0.78347

Spain IBS	Full OTUs	Bray-Curtis	44	2.590626	26	3.310806	0.782476
